# Notch Signaling Pathway Is Involved in bFGF-Induced Corneal Lymphangiogenesis and Hemangiogenesis

**DOI:** 10.1155/2019/9613923

**Published:** 2019-08-20

**Authors:** Fang Xie, Xue Zhang, Wenting Luo, Hongyan Ge, Dawei Sun, Ping Liu

**Affiliations:** ^1^Department of Ophthalmology, The First Affiliated Hospital of Harbin Medical University, Harbin, China; ^2^The Key Laboratory of Myocardial Ischemia, Chinese Ministry of Education, Harbin, China; ^3^Department of Ophthalmology, The Second Affiliated Hospital of Harbin Medical University, Harbin, China

## Abstract

**Background:**

Notch/Dll4 involvement in cornea neovascularization (CRNV) and lymphangiogenesis is unclear. This study aimed to explore the role of notch signaling in basic fibroblast growth factor- (bFGF-) induced corneal lymphangiogenesis and hemangiogenesis.

**Methods:**

Corneal stroma of C57BL/6 mice was implanted with bFGF- or phosphate-buffered saline- (PBS-) soaked pellets. Corneal lymphangiogenesis and neovascularization were evaluated by immunofluorescence. Vascular endothelial growth factor-A (VEGF-A), Delta-like ligand 4 (Dll4), and Notch1 mRNA and protein expression were examined on days 1, 3, 7, and 14 by real-time polymerase chain reaction and western blot. Corneal cells were treated with ranibizumab, dexamethasone, and *γ*-secretase inhibitor (GSI). Microspheres were used to evaluate corneal hemangiogenesis in vivo.

**Results:**

Corneal hemangiogenesis reached its peak on day 7 after bFGF implantation, and corneal lymphangiogenesis was significantly higher on day 7 and 14, compared with PBS. mRNA and protein expression of VEGF-A, Dll4, and Notch1 were higher in bFGF-induced animal models compared with controls. Corneal hemangiogenesis and lymphangiogenesis decreased after 7 days of ranibizumab or dexamethasone treatment. After adding GSI for 24 h in bFGF-induced cells, the expression of Notch1 and Dll4 were downregulated compared with that in the control group whereas the expression level of VEGF-A was upregulated. Fluorescent particle number was higher in the GSI group. Ranibizumab and dexamethasone decreased the fluorescence signal.

**Conclusion:**

The notch signaling pathway plays a role in regulating VEGF expression, affecting corneal lymphangiogenesis and hemangiogenesis in mice. The molecular imaging probe technique can visualize the changes in the VEGF-A expression level of corneal limbus hemangiogenesis.

## 1. Introduction

The normal cornea maintains a transparent state without blood or lymphatic vessels through a natural imbalance in favor of anti-angiogenesis factors against pro-angiogenesis factors [1, 2]. Since the corneal limbus is rich in both blood and lymphatic vessels, immune responses, trauma, infections, or surgery can induce the capillaries in the corneal limbus to grow into the central area of cornea, that is cornea neovascularization (CRNV) and lymphangiogenesis [1, 2]. These processes lead to cornea opacities, seriously affecting vision [1, 2]. The molecular mechanisms of the regulation of CRNV and lymphangiogenesis are not fully understood, and it is necessary to understand this disease better. In this study, we mean to explore the mechanism of corneal hemangiogenesis and lymphangiogenesis, focus on corneal vascularization, and regulate the Notch signaling pathway to induce the increase or decrease expression of hemangiogenesis and lymphangiogenesis.

Hemangiogenesis and lymphangiogenesis are regulated by the members of vascular endothelial growth factor (VEGF) family through a complex process and a dynamic balance between pro-angiogenesis and anti-angiogenic factors [3, 4]. VEGF stimulates neovascularization by promoting endothelial cell proliferation and migration, enhancing proteolytic activity, increasing vascular permeability, and inducing capillary lumen formation [5]. VEGF-A can stimulate corneal hemangiogenesis and lymphangiogenesis in a dose-dependent manner [6, 7]. Targeting VEGF and its receptors is an important therapeutic approach for many neovascular diseases [8, 9], but VEGF is the only factor promoting neovascularization [10, 11].

The Notch signaling pathway plays a key role in embryonic vascular development, adult angiogenesis, and vascular homeostasis [12, 13]. It interacts with the VEGF pathway: VEGF can induce the Notch pathway, and the Notch pathway can also induce VEGF through feedback, maintaining the signal intensity to maintain angiogenesis [14, 15]. Delta-like ligand 4 (Dll4) is a ligand of Notch and plays an important role in vascular development and homeostasis [14, 16]. Dll4 plays a key role in choroidal neovascularization (CNV) [17]. Nevertheless, the involvement of Notch/Dll4 pathway in CRNV and lymphangiogenesis is still unclear.

Therefore, the aim of this study was to explore the role of Notch signaling pathway in basic fibroblast growth factor- (bFGF-) induced corneal lymphangiogenesis and hemangiogenesis. Ranibizumab and dexamethasone have been reported to suppress hemangiogenesis and lymphangiogenesis in the cornea [18, 19]. To do so, models of CRNV and lymphangiogenesis were established using stimulating corneal bFGF pellets [20, 21]. The changes in the expression level of Dll4 and interrelationship with VEGF were observed to determine the specific mechanisms of the involvement of VEGF and Notch in CRNV.

## 2. Materials and Methods

### 2.1. Animals

Specific pathogen-free male C57BL/6 mice (8 weeks old, weighing 22–26 g; Beijing Weitong Lihua Experimental Animal Technology Co., Ltd., Beijing, China) were housed in a clean facility with free access to water and food and submitted to a 12 h light/dark cycle. All animal experiments were approved by the Animal Ethics Committee of Harbin Medical University.

### 2.2. Preparation of bFGF and Phosphate-Buffered Saline Pellets

Absolute ethanol (500 *μ*L) was used to dissolve 60 mg of Hydron, followed by shaking for 10 min. The solution was kept at room temperature for 12 h until completely dissolved. Sucralfate (10 mg) and 25 *μ*L of bFGF or the same volume of phosphate-buffered saline (PBS) were placed in a centrifuge tube and completely dissolved. Hydron (10 *μ*L) was added to the tubes containing PBS and bFGF and fully mixed. The mixture was then spread on a 15 × 15 grid nylon mesh with a pore size of 340 *μ*m to prepare a pellet containing bFGF at a concentration of 100 ng/grid. After drying at room temperature for 30 min, the pellets were collected and stored at −20°C.

### 2.3. Establishment of the Animal Model of CRNV and Lymphangiogenesis

The mouse model of CRNV was established as previously described [20, 21]. The mice were anesthetized with an intraperitoneal injection of pentobarbital sodium (80 mg/kg). The bFGF pellets were implanted in the corneal limbus within 1 mm of the corneal stroma. The control group was implanted with PBS pellets. After the operation, erythromycin eye ointment was administered. The eyes were directly observed and photographed under a microscope (Olympus DF PLAPO 1X-4, Olympus, Tokyo, Japan) on postoperative days 1, 3, 7, and 14. The mice were divided into control, bFGF (model), treatment, and GSI groups. Immediately after modeling, the mice in the treatment group (*n* = 20) were administered with one eye drop (5 *μ*L/drop, at the concentration of 5 mg/mL) of ranibizumab/dexamethasone, four times a day for 7 days, until tissue harvesting. The treatment groups were subdivided as follows: PBS 7 days control + dexamethasone; PBS 7 days control + ranibizumab; bFGF 7 days model + dexamethasone; and bFGF 7 days model + ranibizumab. The mice in the GSI group (*n* = 10) were implanted with bFGF pellets and then injected with 10 mg/kg GSI intraperitoneally on postoperative day 2, twice a day for 5 days, until tissue harvesting on day 7. The mice in the GSI-control group were injected intraperitoneally with 1 mL/kg normal saline instead of GSI.

### 2.4. Culture of Human Umbilical Vein Endothelial Cells

Human umbilical vein endothelial cells (HUVECs) were purchased from the American Type Culture Collection (ATCC; VA, USA) and cultured in Dulbecco's modified Eagle's medium (Gibco, Invitrogen Inc., CA, USA) supplemented with 10% fetal bovine serum (Gibco), 100 U/mL penicillin, and 100 mg/mL streptomycin. The cells were incubated at 37°C in a humidified chamber supplemented with 5% CO_2_. HUVECs at passages 3–5 were used for the subsequent experiments. HUVECs were subjected to dexamethasone (0.003 *μ*mol/mL), GSI (0.003 *μ*mol/mL), or ranibizumab (60 *μ*g/mL) treatment.

### 2.5. Immunofluorescence

After anesthesia with an intraperitoneal injection of pentobarbital sodium (80 mg/kg), the eyes were completely collected and fixed in 4% paraformaldehyde (PFA) for 30 min. The cornea was cut along the corneal limbus for full cornea mounting. The specimens were embedded and fixed with optimal cutting temperature compound (OCT) tissue freezing medium OCT compound (Sakura, Torrance, USA) and cut into 10 *μ*m sections. Rat anti-mouse CD31 (5 *μ*g/mL; Pharmingen, BD Biosciences, NJ, USA) and rabbit anti-mouse LYVE-1 (4 *μ*g/mL; Abcam, Cambridge, UK) antibodies were used to label the corneal hemangiogenesis and lymphangiogenesis. The sections were incubated overnight at 4°C and washed thrice with PBS (10 min each time). The sections were incubated for 1 h with fluorescence secondary goat anti-rabbit Alexa Fluor 647 immunoglobulin G (IgG) (20 *μ*g/mL) and goat anti-rat Alexa Fluor 488 IgG (20 *μ*g/mL). The sections were again washed thrice with PBS (10 min each time). A mounting medium (Lab Vision, Thermo Fisher Scientific, MA, USA) was used to seal the sections. Fluorescence was observed under a fluorescence microscope (Leica DM400B, Leica Microsystems, Ltd., Wetzlar, Germany). ImageJ (1.50i; National Institutes of Health, MD, USA) was used with standardized illumination and contrast.

### 2.6. Western Blot

Four corneal tissues in each group were cut into fragments of 1 × 1 mm^2^ and placed in 200 *μ*L of RIPA lysis buffer (Invitrogen Inc., CA, USA) for 10–15 min. The tissues were treated with ultrasound on ice for 2 s at 60% of ultrasound energy (intervals of 10 min for a total of three treatments). The tissues were incubated on ice for 30 min, followed by centrifugation at 13,000 rpm for 15 min at 4°C. The bicinchoninic acid method was used to determine the protein concentration. The proteins were separated on 10% sodium dodecyl sulfate-polyacrylamide gel electrophoresis (SDS-PAGE) (60 *μ*g/well) and transferred onto a polyvinylidene difluoride membrane. The membrane was placed in 5% milk blocking buffer at 37°C for 1 h and then incubated with primary antibodies against rabbit anti-mouse Notch1 (1 : 1000; Abcam, Cambridge, UK), rabbit anti-mouse Dll4 (1 : 1000; Abcam), and rabbit anti-mouse VEGF-A (1 : 1000; Abcam) overnight at 4°C. The membranes were washed thrice with PBS (10 min each time). The membranes were incubated with a secondary antibody goat anti-rabbit IgG (H + L) (1 : 5000; ZSGB-BIO, Beijing, China) for 1 h. Proteins were detected using enhanced chemiluminescence staining and washed thrice with PBS (10 min each time). Anti-beta actin (1 : 1000; Abcam) was used as the internal control. Proteins were detected using ECL (Applygen, Beijing, China) staining and quantified using an automatic chemiluminescence imaging analysis system (Tanon-5200, Shanghai, China). Each experiment was repeated three times.

### 2.7. Real-Time Polymerase Chain Reaction

The total corneal RNA was extracted using Trizol (Invitrogen Inc). The RNA concentration was measured by ultraviolet spectrophotometry (BioSpec-nano, Tokyo, Japan). The RNA was reverse transcribed into cDNA using cDNA reverse transcription kits (Takara Bio, Otsu, Japan), and the expression of mRNA in each sample was detected by real-time polymerase chain reaction (PCR). PCR primers were designed according to GenBank sequences (Table 1). The semiquantitative analysis of the intensity of each PCR product was performed and normalized against mRNA levels of glyceraldehyde-3-phosphate dehydrogenase (GAPDH) as a housekeeping gene, based on the 2^−ΔΔCT^ method. The experiments were performed in triplicate.

### 2.8. Molecular Imaging Probe

Carboxylated fluorescent or nonfluorescent microspheres (MSs) (2 *μ*m, Polysciences, Inc., PA, USA) were covalently conjugated to protein G (Sigma, MO, USA), using a carbodiimide coupling kit (Polysciences, Inc., PA, USA). VEGF-A antibody was incubated with MSs at 0.4 mg/mL overnight at room temperature. The MSs were washed in PBS with 1% bovine serum albumin before being used in vivo. The fluorescent MSs (6 × 10^7^) were injected into each mouse. After systemic injection, the interactions of these MSs with the endothelium of normal and angiogenic vessels of live animals were studied by intravital video microscopy [22].

### 2.9. Statistical Analysis

All values were expressed as mean ± standard error of the mean. Data were analyzed using one-way analysis of variance and Student's post hoc test. Two-tailed *P* values <0.05 were considered statistically significant. All analyses were performed using SPSS 19.0 (IBM, NY, USA).

## 3. Results

### 3.1. bFGF-Induced CRNV and Lymphangiogenesis

**Figure 1:**
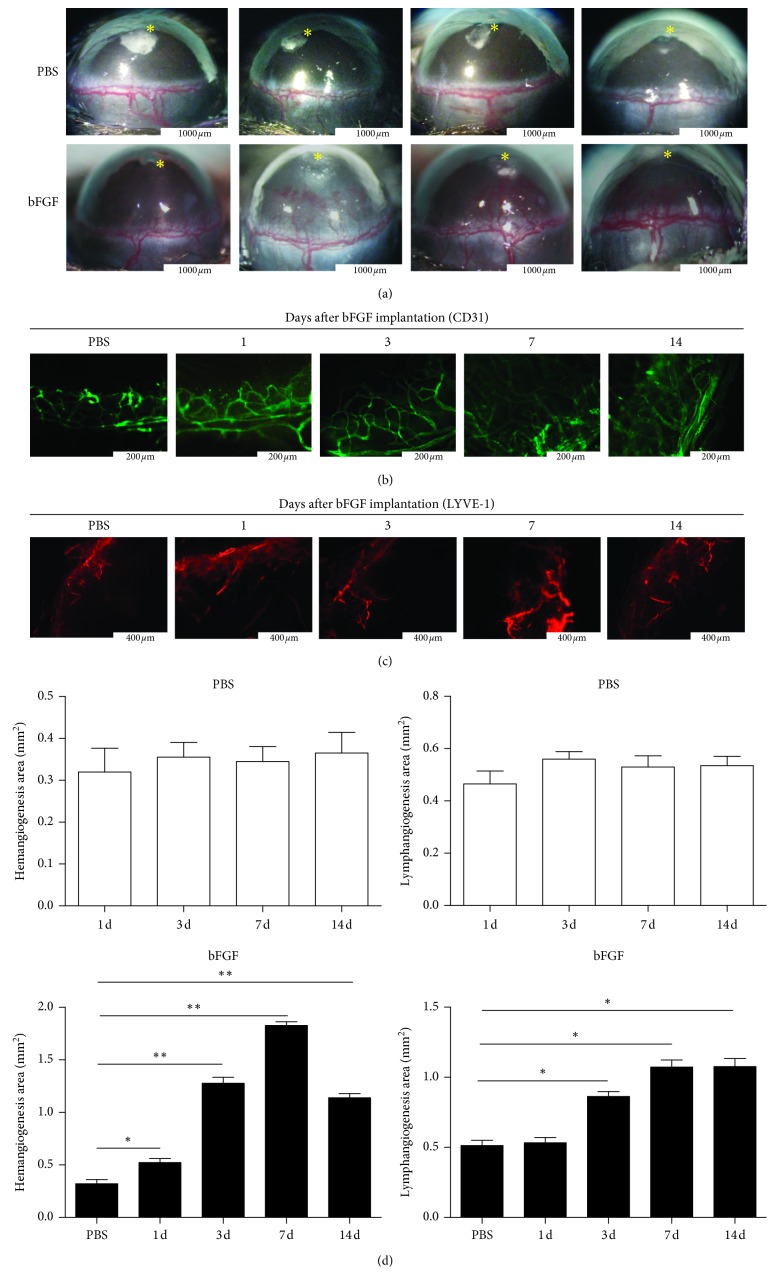
bFGF-induced lymphangiogenesis and hemangiogenesis. (a) Control group: microscopic pictures of corneal neovascularization were examined on days 1, 3, 7, and 14 after PBS pellet implantation (*P* > 0.05). bFGF group: microscopic pictures of corneal neovascularization were examined on days 1, 3, 7, and 14 after bFGF pellet implantation (*P* < 0.05) (bar, 1000 *μ*m). ^*∗*^Sites of pellet implantation. (b) bFGF group: the staining of corneal flat mounts for hemangiogenesis (CD31, green) were examined on days 1, 3, 7, and 14 after bFGF or PBS pellet implantation (*P* < 0.05) (bar, 200 *μ*m). (c) bFGF group: the staining of corneal flat mounts for lymphangiogenesis (LYVE-1, red) was examined on days 1, 3, 7, and 14 after bFGF or PBS pellet implantation (*P* < 0.05) (bar, 400 *μ*m). (d) Quantitative analysis of lymphangiogenesis and hemangiogenesis on the indicated days after PBS implantation (*n* = 4–6) (*P* > 0.05). Quantitative analysis of lymphangiogenesis and hemangiogenesis on the indicated days after bFGF implantation (*n* = 5–8). ^*∗*^*P* < 0.05; ^*∗∗*^*P* < 0.01.

The formation of CRNV was observed by microscopic imaging on days 1, 3, 7, and 14 after PBS and bFGF implantation. The changes in corneal hemangiogenesis after implanting the PBS pellets were not statistically significant (Figures 1(a) and 1(d)). On the first day after implanting the bFGF pellets in the model group, neovascularization was found at the corneal limbus. On the third day, CRNV increased and grew to the corneal center. The CRNV peaked on day 7 when neovascularization could reach the pellet but subsided on day 14 (Figures 1(a) and 1(d)).

LYVE-1 was used to label the lymphatic endothelial cells and detected by immunofluorescence and corneal frozen sections. The corneal lymphangiogenesis was located near the vascular rings of the corneal limbus in the PBS control group, and the tip of the lymphangiogenesis was round. The new lymphangiogenesis in the model group were grown from the tip of the normal lymphangiogenesis and extended outward; they were observed on the third day after bFGF-induced modeling and highly expressed from day 7 to day 14. The results showed that the new lymphangiogenesis in the bFGF model group was significantly higher in number compared with those in the PBS control group on day 14 (Figure 1(c)).

CD31 was used to label the vascular endothelial cells and detected by immunofluorescence. In the model group, neovascularization was observed on the first day after bFGF implantation and gradually increased with time. When CRNV reached its peak on day 7, neovascularization gradually decreased and the new hemangiogenesis began to subside on day 14. These changes were consistent with the microscopy results (Figure 1(b)).

### 3.2. Increased mRNA and Protein Expression Levels of VEGF-A, Dll4, and Notch1 in bFGF-Induced Animal Models

**Figure 2:**
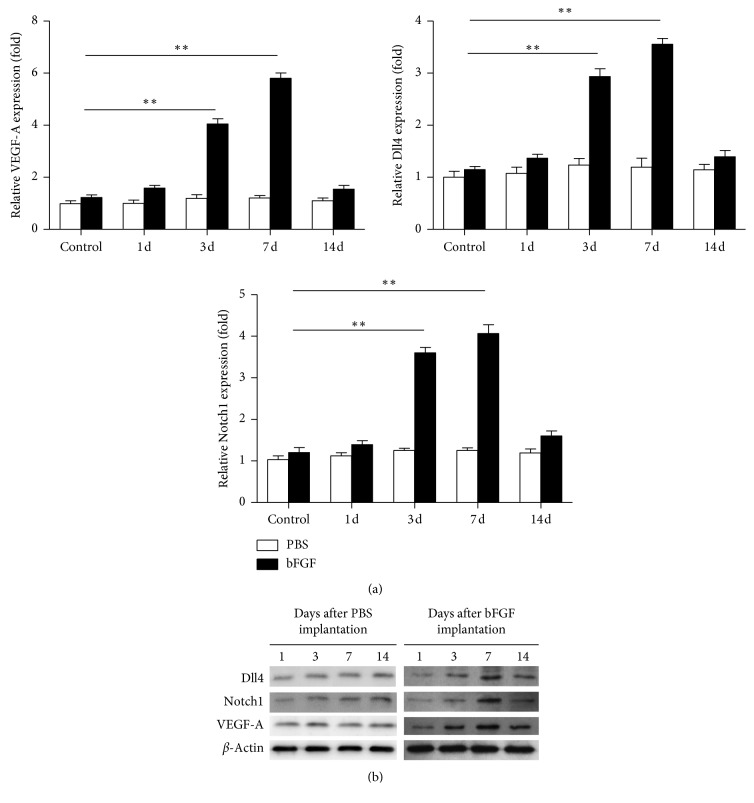
(a) VEGF-A, Notch1, and Dll4 mRNA expression levels on the indicated days in the corneal micropocket assay after PBS stimulation and bFGF stimulation were similar; ^*∗∗*^*P* < 0.01. (b) Representative western blots for VEGF-A, Notch1, and Dll4 from PBS- or bFGF-implanted corneas (days 1, 3, 7, and 14).

Real-time polymerase chain reaction (RT-PCR) showed that the mRNA expression levels of VEGF-A, Dll4, and Notch1 mRNA were lower in the PBS control group but significantly upregulated in the bFGF-induced model group on days 1, 3, and 7 (Figure 2(a)). Their peak was reached on day 7, and they decreased gradually thereafter. Their mRNA expression level was slightly higher than that in the PBS control group on day 14, but the differences were not significant. The changes in the expression levels of VEGF-A, Dll4, and Notch1 were consistent with the changes in CRNV and lymphangiogenesis (Figure 2(a)). The expression level and trend of VEGF-A, DLL4, and Notch1 proteins detected by western blot were consistent with those of RT-PCR (Figure 2(b)).

### 3.3. Ranibizumab Significantly Reduced bFGF-Induced CRNV and Lymphangiogenesis

**Figure 3:**
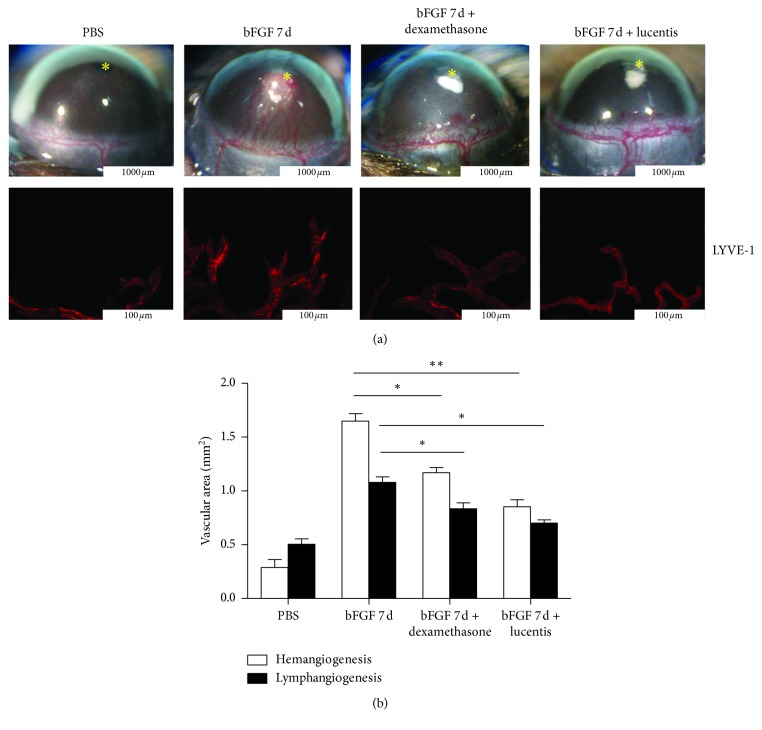
Expression of lymphangiogenesis- and hemangiogenesis-associated factors. (a) Corneal hemangiogenesis and lymphangiogenesis (LYVE-1, red) were reduced by dexamethasone and ranibizumab, but ranibizumab had a stronger inhibitory effect (bar, 1000 *μ*m; 100 *μ*m) ^*∗*^Sites of pellet implantation. (b) Quantitative analysis of lymphangiogenesis and hemangiogenesis in the treatment group (*n* = 4–6). ^*∗*^*P* < 0.05; ^*∗∗*^*P* < 0.01.

Since CRNV and lymphangiogenesis had their peak on day 7 after bFGF modeling, day 7 of the bFGF-induced model was selected as the observation time in the treatment group. No significant changes were observed in corneal hemangiogenesis among the groups (PBS normal control, PBS normal control + dexamethasone, and PBS normal control + ranibizumab). In the bFGF model + dexamethasone group (5 *μ*L/drop; conc.: 5 mg/mL, four times daily for 7 days), neovascularization after dexamethasone treatment was lower than that in the bFGF model control group. After ranibizumab treatment (5 *μ*L/drop; conc.: 5 mg/mL, four times daily for 7 days), neovascularization largely subsided, indicating that the treatment effect of ranibizumab was better than that of dexamethasone (Figures 3(a) and 3(b)).

### 3.4. Ranibizumab Significantly Reduced the Expression Levels of VEGF-A, Dll4, and Notch1 in bFGF-Induced Animal Models

**Figure 4:**
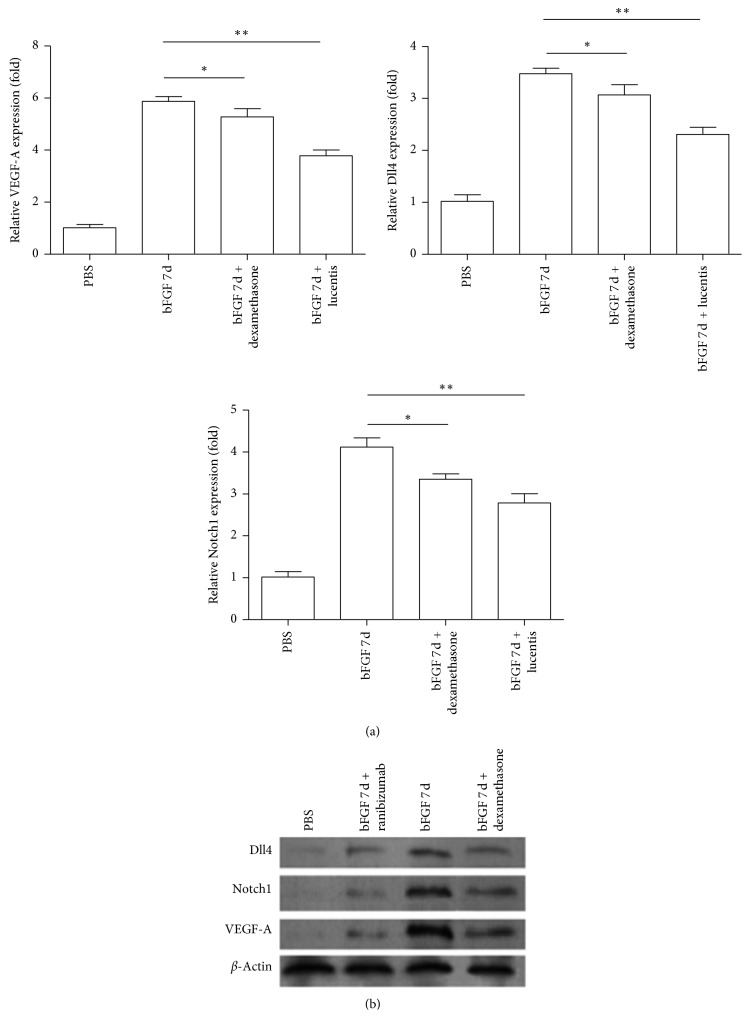
Relative expression of VEGF-A, Notch1, and Dll4 mRNA in the treatment group. (a) Dexamethasone and ranibizumab inhibited the relative expression of VEGF-A, Notch1, and Dll4 mRNA (*n* = 4–6). ^*∗*^*P* < 0.05; ^*∗∗*^*P* < 0.01. (b) Representative western blot for VEGF-A, North1, and Dll4 in the treatment group.

The effects of dexamethasone and ranibizumab on VEGF-A, Dll4, and Notch1 were further assessed. The mRNA and protein levels of VEGF-A, Dll4, and Notch1 in the bFGF model + dexamethasone group were significantly lower than those in the bFGF model + control group (*P* < 0.05), whereas the mRNA and protein expression levels of VEGF-A, Dll4, and Notch1 in the bFGF model + ranibizumab group were significantly lower than those in the bFGF model + control group (*P* < 0.01) but still higher than those in the PBS normal control group (Figures 4(a) and 4(b)).

### 3.5. GSI Upregulated VEGF-A Expression Level in bFGF-Induced HUVEC Cell Models

**Figure 5:**
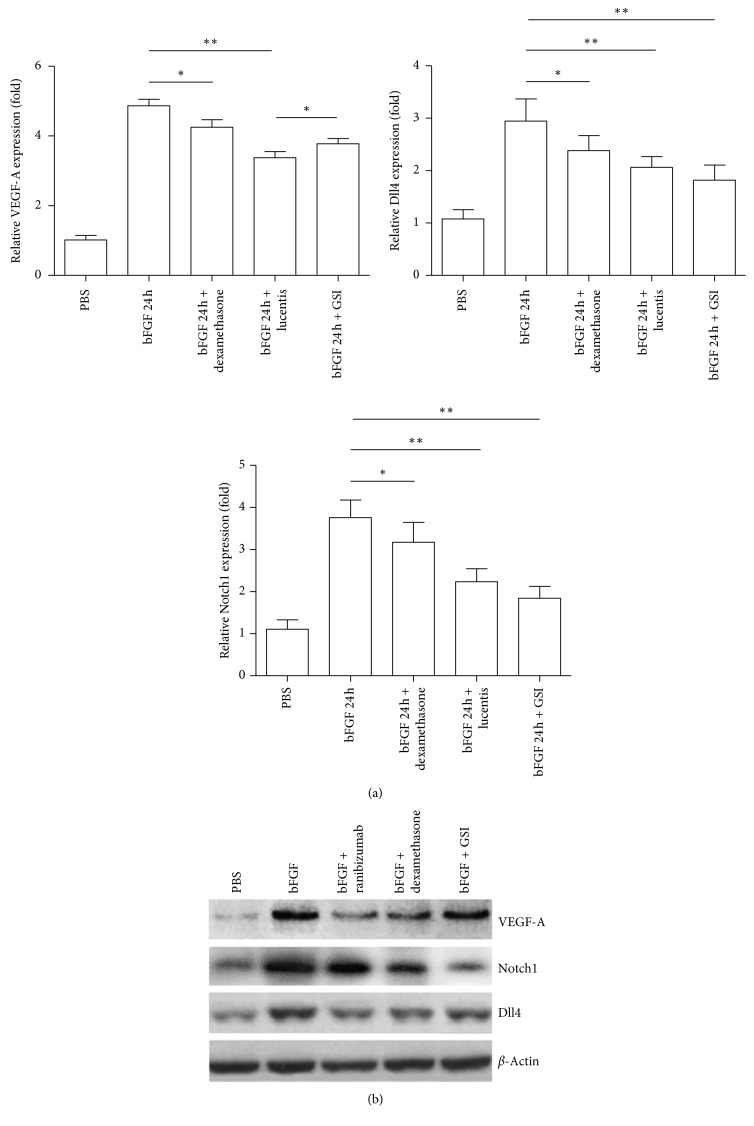
(a) VEGF-A, Notch1, and Dll4 mRNA expression. GSI inhibited the expression of Notch1 and Dll4 mRNA and promoted the Abs-conjugated MS in corneal vessels of treated, untreated, and bFGF-implanted eyes (day 7; *n* = 5). ^*∗*^*P* < 0.05; ^*∗∗*^*P* < 0.01. (b) Western blot analysis of VEGF-A, Notch1, and Dll4 protein expression.

In vitro cell experiments were performed to validate further the phenomenon observed in vivo. In the bFGF-induced cell model, the gene and protein expression levels of VEGF-A, Notch1, and Dll4 were detected after ranibizumab or dexamethasone treatment. The results were consistent with the in vivo results. The expression levels of VEGF-A, Notch1, and Dll4 were downregulated by ranibizumab (Figure 5(a)), but it was unclear whether the Notch pathway could feedback the expression of VEGF-A. After culturing bFGF-induced cells with GSI for 24 h, the expression level of Notch1 and its ligand Dll4 was downregulated compared with that of the control group, while the expression level of VEGF-A increased (Figures 5(a) and 5(b)).

### 3.6. GSI Promoted bFGF-Induced CRNV and Increased Neovascularization

**Figure 6:**
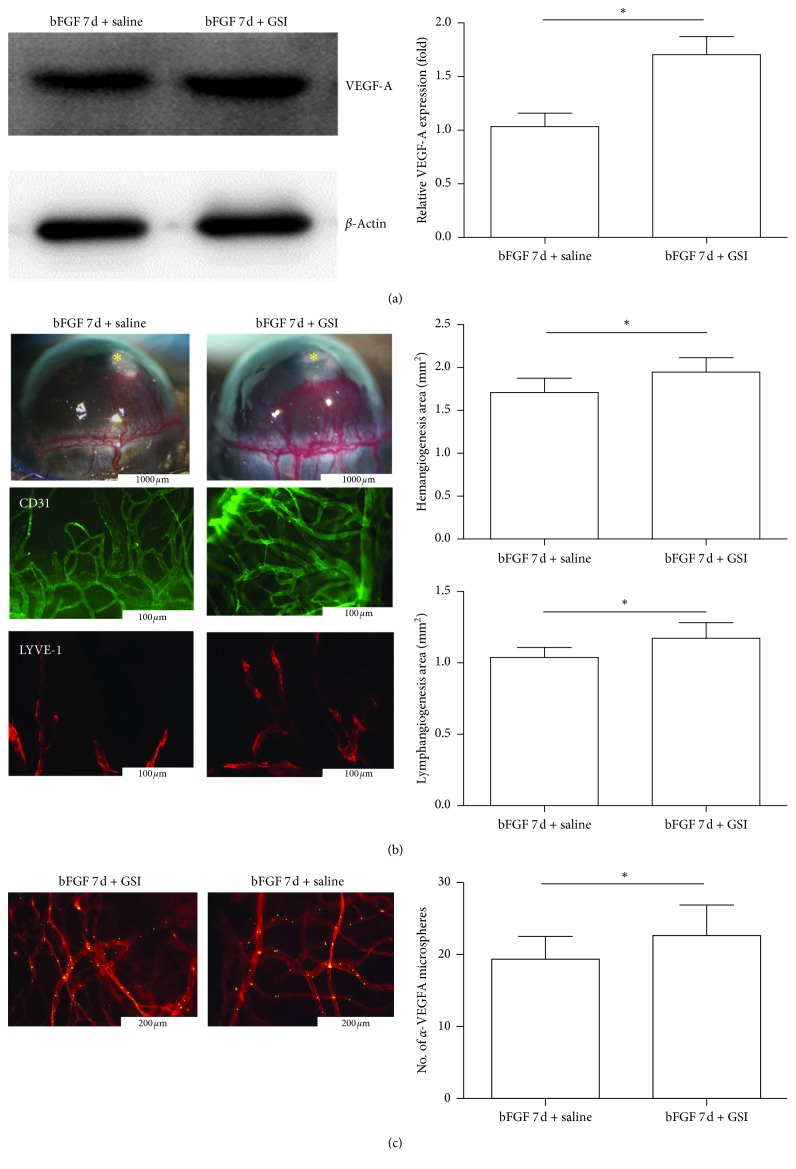
Vascular areas of lymphangiogenesis and hemangiogenesis in the GSI groups. (a) Representative western blot for VEGF-A in the GSI group; ^*∗*^*P* < 0.05. (b) Quantitative analysis of lymphangiogenesis and hemangiogenesis in the GSI group (*n* = 5); ^*∗*^*P* < 0.05 (bar, 1000 *μ*m; 100 *μ*m). ^*∗*^Sites of pellet implantation. (c) *α*-VEGF-As mAb-conjugated microspheres (green) and rhodamine-conjugated conA (red) in GSI and GSI-control groups (day 7; *n* = 5) (^*∗*^*P* < 0.05; bar, 200 *μ*m).

After the 7-day administration of GSI in a bFGF-induced mouse model, western blot was used to detect VEGF-A expression, and corneal immunofluorescence was used to detect the changes in the area of lymphangiogenesis and hemangiogenesis. GSI-treated CRNV could increase the expression level of VEGF-A and had more lymphatic and neonatal vessels compared with the GSI-control group (Figure 6(b)). Microscopic observations revealed that the area of GSI-treated CRNV also increased (Figure 6(c)), indicating that GSI negative feedback could increase the expression level of VEGF-A by inhibiting the activity of the Notch1 pathway (Figure 6(a)).

### 3.7. Molecular Probe Detection

**Figure 7:**
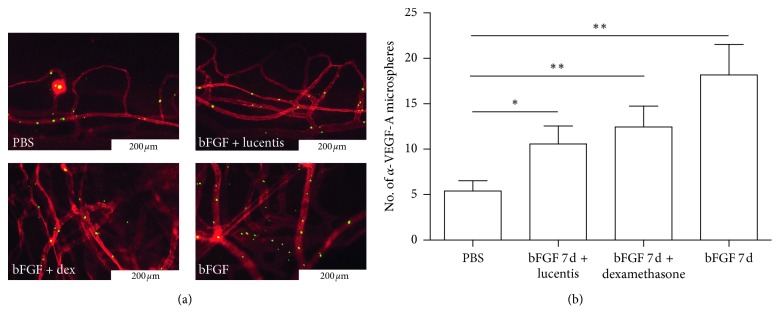
In vivo molecular imaging of *α*-VEGF-A in bFGF-induced hemangiogenesis. (a) *α*-VEGF-As mAb-conjugated microspheres (green) and rhodamine-conjugated conA (red) (bar, 200 *μ*m). (b) Quantitation of the number of *α*-VEGF-A microspheres (*n* = 5–8). ^*∗*^*P* < 0.05; ^*∗∗*^*P* < 0.01.

Fluorescent MSs were observed in the model group on day 7 and in the control group to evaluate the effect of the molecular probe in vivo. A few anti-VEGF fluorescent particles were observed in the control group, but the number of fluorescent particles was significantly higher in the model group (Figure 7(a)). The number of fluorescent particles in the dexamethasone- and ranibizumab-treated groups was significantly lower compared with that in the nontreated group (Figure 7(b)).

## 4. Discussion

The results suggested that the Notch signaling pathway played a role in regulating VEGF expression, affecting corneal lymphangiogenesis and neovascularization in mice. The molecular imaging probe technique could visualize the changes in the VEGF-A-upregulated expression level of corneal limbus hemangiogenesis.

The mouse model of CRNV was successfully induced using published techniques [20, 21]. This model was used in the present study to demonstrate that the Notch signaling pathway might participate in bFGF-induced corneal lymphangiogenesis and hemangiogenesis [23]. The results showed that hemangiogenesis and lymphangiogenesis had a time-dependent relationship with the implantation of bFGF pellet. Indeed, the expression levels of VEGF-A, Notch1, and Dll4 progressively increased to reach the peak on day 7 and then decreased. Microscopic observations of new vessels also concurred with the molecular results. It is known that VEGF-A can stimulate corneal hemangiogenesis and lymphangiogenesis in a dose-dependent manner [6, 7]. In addition, VEGF can stimulate the Notch pathway, which in turn can stimulate VEGF-A expression through a feedback loop [14, 15]. Inhibition of VEGF-A expression also downregulated Notch1 and Dll4 gene and protein expression levels. VEGF-A could activate the Notch pathway, but whether Notch can feedback to regulate the expression of VEGF-A is not clear. Our research showed that the inhibition of Notch pathway activity could downregulate the expression levels of Notch1 and Dll4, increase the expression level of VEGF-A by negative feedback as well, and promote bFGF to induce more lymphangiogenesis and hemangiogenesis. These results were in agreement with other studies of Notch inhibition [24, 25].

The results showed that treatment with corticosteroids (dexamethasone) and anti-VEGF drugs (ranibizumab) could effectively inhibit the formation of CRNV; ranibizumab was more effective. The dose for each treatment method was determined by the previous report [18]. Ranibizumab could significantly decrease the mRNA and protein expression levels of VEGF-A, Dll4, and Notch1 in the bFGF-induced model, highlighting the roles of VEGF-A, Dll4, and Notch1 in CRNV and lymphangiogenesis. Indeed, ranibizumab is known to have a potential to treat CRNV [26, 27]. The present study suggested that ranibizumab could degrade corneal vascularization, and it could be used in CRNV treatment. However, lymphatic vessels also play a crucial role in the induction of a rejection reaction against the corneal graft [28]. Thus, the present study provided a new idea by which antilymphangiogenic growth improved transplant survival.

The molecular imaging probe can help in studying early changes in the expression levels of some relevant molecules of microvascular endothelial cells in vivo under physiological conditions [29, 30]. Moreover, it can display the abnormal expression of surface adhesion molecules in endothelial cells in vivo. After molecular probes are injected into the systemic circulation, their interactions produce rolling or adhesion based on the expression levels of surface adhesion molecules in vascular endothelial cells. In the present study, the expression levels of VEGF-A in the bFGF model group was significantly higher than that in the normal PBS model group. The conclusions drawn from this approach supported the results of the previous studies about the role of VEGF-A in neovascularization-related diseases [6, 14, 24, 31, 32]. Although molecular imaging is still a research tool and is not used clinically, this technique is useful in visualizing the changes in the molecular level of new corneal limbus hemangiogenesis. In vivo molecular probe imaging has great potential to be able to identify more clearly the severity of the disease compared with direct examination.

In conclusion, the Notch signaling pathway plays a role in regulating VEGF expression, affecting corneal lymphangiogenesis and neovascularization in mice. VEGF inhibition could be used for CRNV treatment. Only a limited panel of genes and proteins was examined, and a larger panel should be studied to grasp a better understanding of the mechanisms involved in CRNV.

## Figures and Tables

**Table 1:** Primers for RT-PCR.

Primer	Sense	Sequence (5′–3′)	Fragment size (bp)
GAPDH	Sense	GCGCTGAGTACGTCGTGGAG	196
	Antisense	CAGTTGGTGGTGCAGGAGG	
VEGF-A	Sense	GCACCCATGGCAGAAGGA	156
	Antisense	CACACAGGATGGCTTGAAGATG	
Dll4	Sense	GCCCTTCAATTTCACCTGGC	157
	Antisense	CAATAACCAGTTCTGACCCACAG	
Notch1	Sense	GAGGCGTGGCAGACTATGC	140
	Antisense	CTTGTACTCCGTCAGCGTGA	

## Data Availability

The data used to support the findings of this study are available from the corresponding author upon request.

## Abbreviations

CRNV: Cornea neovascularization
bFGF: Basic fibroblast growth factor
PBS: Phosphate-buffered saline
VEGF: Vascular endothelial growth factor
Dll4: Delta-like ligand 4
CNV: Choroidal neovascularization
HUVECs: Human umbilical vein endothelial cells
PFA: Paraformaldehyde
OCT: Optimal cutting temperature compound
SDS-PAGE: Sulfate-polyacrylamide gel electrophoresis
MSs: Microspheres
RT-PCR: Real-time polymerase chain reaction
GSI: *γ*-Secretase inhibitor.
